# Antimicrobial Usage in Smallholder Poultry Production in Nigeria

**DOI:** 10.1155/2022/7746144

**Published:** 2022-02-28

**Authors:** Oladeji Bamidele, Tunde A. Amole, Oluwafikayo A. Oyewale, Olayinka O. Bamidele, Abdulmojeed Yakubu, Uduak E. Ogundu, Folasade O. Ajayi, Waheed A. Hassan

**Affiliations:** ^1^International Livestock Research Institute (ILRI), P.M.B. 5320, Ibadan 200001, Nigeria; ^2^Department of Biological Sciences, Kings University, Odeomu 220104, Nigeria; ^3^African Centre of Excellence for Genomics of Infectious Diseases, Redeemer's University, Ede, Nigeria; ^4^Faculty of Medicine, Hebrew University, Jerusalem, Israel; ^5^Department of Community Medicine, UNIOSUN Teaching Hospital, Osogbo, Osun State, Nigeria; ^6^Department of Animal Science, Faculty of Agriculture, Nasarawa State University, Keffi, Shabu-Lafia Campus Lafia, Nasarawa State, Nigeria; ^7^Department of Animal Science and Technology, Federal University of Technology, Owerri, Imo State, Nigeria; ^8^Department of Animal Science, University of Port-Harcourt, Choba, Rivers State, Nigeria; ^9^Department of Animal Science, Usmanu Danfodiyo University, Sokoto, Sokoto State, Nigeria

## Abstract

The indiscriminate use of antimicrobials in livestock production is of increasing concern due to the threat of antimicrobial resistance in both humans and animals. Much emphasis has been placed on intensively managed poultry production systems, which routinely use antimicrobials as against smallholder poultry production systems (SPPS). Therefore, this study investigated the use of antimicrobials among smallholder poultry farmers in Nigeria, and compared the prevalence of antimicrobial drug use against the practice of ethnoveterinary medicine (EVM). A cross-sectional study was conducted in five states (agroecologies) of Nigeria using structured questionnaires administered on a total of 350 farmers. The practice of EVM was prevalent among most of the farmers (39%). The western method (pharmaceuticals) was practiced by a large proportion of farmers (60%), either solely (25%) or in combination with EVM (35%). Antimicrobials were used primarily for treatment and prevention of diseases (78%). Semi-scavenging system of production had the highest proportion (49%) of farmers using antimicrobials, compared to semi-intensive (37%) and scavenging (14%) systems. Gender (*χ*^2^ = 9.30, *p* = 0.01), and location (*χ*^2^ = 216.86, *p* ≤ 0.001), influenced farmers' choice of methods for bird treatment. Education (odds ratio [OR] odds ratio [OR] 3.06, 95% CI 2.10–4.44), income (OR 1.99, 95% CI 1.10–3.59) and management system (OR 1.97, CI% 1.1–3.45) were most associated with antimicrobial use. Critically important antibiotics, with lower to higher risk of antimicrobial resistance, were used by farmers (40%). These findings showed the indiscriminate use of antimicrobials by farmers and the potential risk of antimicrobial resistance within the SPPS in Nigeria.

## 1. Introduction

The use of antimicrobials in livestock production, for disease prevention and growth promotion, is of increasing concern owing to the threat of antimicrobial resistance in both humans and animals [1]. Antimicrobial resistance has been described as one of the biggest threats to humanity, affecting critical areas such as global health, food security and livelihoods [2, 3]. Overdependence on antimicrobials by farmers, and their indiscriminate and inappropriate use are the primary divers of antimicrobial resistance [4–6]. Livestock production under intensive management system is characterized by routine use of antimicrobials, and has been associated with increased antimicrobial resistance, especially in poultry [7]. On the other hand, the use of antimicrobials in scavenging and semi-scavenging production systems of smallholder poultry is generally considered low due to the practice of ethnoveterinary medicine by resource-poor farmers [8, 9]. However, recent studies have observed the increasing use of antimicrobials within the free-range, village or backyard poultry system [4, 10].

Also, the introduction of improved chicken breeds into smallholder poultry production systems (SPPS) as an intervention for increasing food security and livelihoods among rural households in sub-Saharan Africa [11–13] has highlighted the associated high risk of mortality in the flock due to the heterogenous condition of such environments for diseases and infection [14, 15]. In order to prevent this risk, and increase the survivability of the improved chickens, smallholder poultry farmers are exposed to the use of antimicrobials as against adopting improved biosafety and biosecurity measures in reducing the high disease burden within the production environment [10, 16]. Unavailability of veterinarians and animal health workers in rural communities is a contributory factor predisposing smallholder poultry farmers to indiscriminate use of antimicrobials [1, 16].

The improved, tropically adapted chicken breeds (FUNAAB Alpha, Noiler, Kuroiler, Sasso, ShikaBrown) were introduced to smallholder poultry farmers in Nigeria through the African Chicken Genetic Gains project (2015–2019) [11, 17]. These breeds, coupled with the existing local chicken ecotypes are a major source of animal protein (eggs, meat) in the country since smallholder poultry contribute 65–77% of the total chicken production in Nigeria [18]. This huge food resource also presents a potential risk and challenge to food safety due to the abuse of antimicrobials within the production systems. Therefore, the objective of this study was to investigate the use of antimicrobials among smallholder poultry farmers in Nigeria, as well as the prevalence of such use in relation to ethnoveterinary practices within the SPPS.

## 2. Materials and Methods

This study (cross-sectional) was conducted in five states (Rivers, Imo, Kwara, Nasarawa, Kebbi) of Nigeria each representing different agroecologies. The agroecological features of these states have been described by Yakubu et al. [19], and Bamidele and Amole [18]. A total of 350 (70 per state) smallholder poultry farmers participated in the study. The sampling method and inclusion criteria were as described by [18]. All the farmers provided informed consent and the study was approved by the Review Committee of the CGIAR COVID-19 Hub: ILRI Nigeria 2021.

Structured questionnaires were designed to provide information in the following areas: socio-demography, poultry production, disease and health management, and training in animal health. Specifically, the questionnaires elicited information on the knowledge of antimicrobial resistance, perceptions on the risks and implications of antimicrobial use, attitude of farmers towards antimicrobial usage, and the impact of COVID-19 pandemic on the procurement of antimicrobials for animal use. The questionnaires were administered to the farmers at their respective homes by trained field officers, using Google Forms accessed on smartphones. The data collection spanned 14 days (14–28 September, 2021). Data were analysed using descriptive and inferential statistics in IBM-SPSS (version 20). Data visualisations were presented using PAST (version 4.03) and Microsoft Excel (Office 2019). The antimicrobial drugs were classified based on their active ingredients and class [20], AWaRe (Access, Watch, Reserve) group list of antimicrobials [21], and WHO categorisation of importance of antibiotics in human medicine [22]. The official Central Bank of Nigeria's average monthly exchange rate used in the study was NGN 410.7 to 1 USD [23].

## 3. Results

### 3.1. Socio-Demographic Characteristics of Respondents

Table 1 shows that there were significant (*p* < 0.05) variations in the socio-demographic characteristics of the farmers across the five locations. Majority of the respondents were females (71.1%, *n* = 249), compared to the male respondents (28.9%, *n* = 101). There were more women (Kebbi: 77.1%, Nasarawa: 90.0%, Rivers: 71.4%, Kwara: 67.1%) than men (Kebbi: 22.9%, Nasarawa: 10.0%, Rivers: 28.6%, Kwara: 32.9%) in all the states, except Imo (Men and women: 50%). About one-third (30.3%) of the respondents had no formal education while over two-thirds (69.7%) had either primary, secondary or tertiary education. Kebbi State had the highest percentage of respondents (51.4%) without any formal education while all the respondents (70) in Imo State had formal education. More women (89.6%) than men (10.4%) had zero years of schooling (106 respondents) and represents about two-fifths (37.4%) of the female respondents. Respondents aged 48–57, and 18–27 years old had the highest (33.1%) and lowest (3.4%) frequency, respectively. Also, respondents who were aged 48 years old and above were about two-thirds (64.3%) of the total number of study participants. Distribution of respondents' average monthly income shows that majority (60.6%) of the farmers earned below NGN 30,000 ($2.4/day). The distribution of the female respondents according to the monthly earnings was 70.3% (< NGN 30,000), 25.7% (NGN 30,000–50,000), and 4.0% (> NGN 50,000) while that of male respondents was 36.6% (< NGN 30,000), 52.5% (NGN 30,000–50,000) and 10.9% (>NGN 50,000).

### 3.2. Implication and Risks of Antimicrobial Usage

Majority (69.8%) of the farmers did not keep records of drug use (Table 2). Imo State had the highest percentage (78.9%) of farmers who kept records compared to Rivers (0%), Kebbi (4.6%), Nasarawa (22.0%), and Kwara (39.3%) states. Most of the farmers were not aware of the drug residue (94.3%), withdrawal period (92.0%), and shelf-life (expiration date) (55.7%) associated with antimicrobial use. Farmers in Rivers (96.2%) and Imo (98.1%) were more aware of the shelf-life and expiration date of the drugs compared to those in other states (Kebbi: 3.1%, Nasarawa: 17.1%, Kwara: 32.1%). A vast majority (93.9%) of the farmers neither knew if drugs consumed by chickens pass to chicken products nor if the drug residues in eggs/meat could affect humans. A greater percentage of the farmers (94.8%) did not know if there was any risk in eating/selling eggs or meat during the administration of antimicrobials to the birds, compared with farmers (5.2%) who indicated knowledge of such. A large proportion of the farmers were not aware of the regulations guarding antimicrobial usage (96.2%), and were neither aware of any governing body responsible for controlling its usage (98.2%) in livestock production. Most of the farmers (93.9%) considered loss of flock (bird mortality) as the most important risk to avoid while considering the use of antimicrobials. The farmers were largely unaware (93.4%) of the danger in the misuse and overuse of antimicrobials both to humans and to animals.

### 3.3. Description of Poultry Production and Management Systems of Respondents

The respondents' number of years of raising chickens (farming experience) ranged from 1 to 5 (16.9%), 6–10 (37.1%), 11–20 (29.1%), and over 20 years (16.9%) (Table 3). Imo (55.9%), Kebbi (27.7%), and Kwara (34.3%) had the highest percentage of respondents who had been raising chickens for 0–5, 6–10, and 11–20 years, respectively. Kwara (35.6%) and Nasarawa (30.5%) accounted for about two-thirds (66.1%) of the respondents who had been raising chickens for over 20 years. A vast majority (88.9%) of the farmers had never received any training on general poultry husbandry and just over one-tenth (11.1%) of them had ever received any training, specifically, on animal disease and health management. Location (*χ*^2^ = 10.74, *p* = 0.03), and not gender (*χ*^2^ = 3.65, *p* = 0.06) was significantly associated with respondents' training on animal disease and health management. In addition to local chickens, farmers kept improved (38.3%) and exotic (39.4%) chickens. The management systems practiced by the farmers were scavenging (13.7%), semi-scavenging (49.2%), and semi-intensive (37.1%). Gender (*χ*^2^ = 6.71, *p* = 0.04) and location (*χ*^2^ = 154.54, *p* ≤ 0.001) were significantly associated with the type of management system. The smallholder poultry production characteristics of the farmers varied significantly (*p* < 0.05) across the locations.

### 3.4. Treatment Methods Used by Farmers

The treatment options adopted by farmers for treating their flock were traditional/ethnoveterinary method (39.4%), western method (i.e. use of pharmaceuticals) (25.1%), and a combination of both traditional and western methods (35.4%) (Table 4). Rivers and Kwara states accounted for about one-third (62.3%) of the farmers who only used traditional/ethnoveterinary method while Kebbi accounted for over two-thirds (68.2%) of the farmers who used western method. Imo and Nasarawa states had the highest percentage of farmers (32.3%) who used both traditional and western methods. A vast majority of the farmers in Kebbi (92.9%), Imo (74.3%), and Nasarawa (58.6%) states used pharmaceuticals, either alone or in combination with ethnoveterinary medicines. Gender (*χ*^2^ = 9.30, *p* = 0.01), location (*χ*^2^ = 216.86, *p* ≤ 0.01), breed-type (*χ*^2^ = 155.92, *p* ≤ 0.01) and management system (*χ*^2^ = 25.08, *p* ≤ 0.01) were significantly associated with the methods of disease treatment used by farmers. More female farmers (43.8%) used the traditional method than the other methods (Western: 21.3%, Both Western and Traditional: 34.9%). Most of the male farmers (36.6%) used both traditional and western methods as against either the traditional (28.7%) or western (34.7%) methods. The primary reason for using the traditional method by most farmers in Nasarawa (34.5%), Rivers (43.2%) and Kwara (40.5%) states was affordability. Most farmers in both Kebbi (100%) and Imo (61.1%) states indicated the ease of availability and accessibility as their primary reason. Most of the farmers identified affordability and availability/accessibility of ethnoveterinary medicines as the primary (38.4%) and secondary (42.8%) reasons for the use of the traditional method.

### 3.5. Characteristics of Farmers' Use of Antimicrobials

A large proportion (81.6%) of farmers who indicated using western method alone or in combination with the traditional method, considered the cost implication of using antimicrobials on their farm.  Table 5 shows that the persons who primarily influence the use of antimicrobials by farmers were veterinarian/animal health workers (27.4%), followed by farmers' own experiences (16.0%), local merchants of day-old and brooded chicks (15.1%), farmer groups (12.7%), extension agents (12.3%), neighbours and friends (10.9%), feed/drug seller (5.2%), and offtakers (0.5%). About two-thirds (63.7%) of the farmers signified the influence of neighbours and friends (22.6%), veterinarian/animal health workers (20.8%), and own experiences (20.3%) as the secondary reasons for the use of antimicrobials. Over half (57.1%) of the farmers indicated “the moment the birds begin to show signs and symptoms of any disease” as the most important factor influencing the use of antimicrobials. A vast majority (78.3%) of the farmers used antimicrobials for both prevention and treatment. Some of the farmers obtained their prescription from extension agents (51.4%) and veterinarians/animal health worker (15.1%) while one-third (33.5%) use self-prescription. The farmers purchased the antimicrobials from feedstores (14.6%), local vendors (30.2%), pharmacies/chemists (21.2%), and veterinary stores (34.0%). Majority (95.5%) of the farmers whose sourcing of antimicrobials was impacted by COVID-19 pandemic were from Kebbi (91.0%) and Nasarawa (61.0%) states. The main routes of drug administration by the farmers were water (52.8%), food (4.3%), food and water (42.9%). Over two-thirds (68.9%) of the farmers used only one type of antimicrobials at a time compared to about one-third (31.1%) of the farmers who used at least two types. Gender (*χ*^2^ = 5.64, *p* = 0.02) and location (*χ*^2^ = 61.93, *p* ≤ 0.001) significantly influenced the pattern of antimicrobials usage. More women used one type (71.2%) or at least two types (54.6%) of antimicrobials compared to men (1 : 28.8%, ≥2 : 45.4%).

Kwara (22.7%) and Imo (51.5%) accounted for most (74.2%) of the farmers who used more than two types of antimicrobials. Farmers used antimicrobials occasionally (infrequent/irregular intervals) (87.7%), seldomly (i.e. rarely, when the local medicines do not seem to be effective) (3.3%), and regularly (once per week/month) (9.0%). Most (84.2%) of the farmers in Kwara State used antimicrobials more regularly than the other states. Gender (*χ*^2^ = 6.30, *p* = 0.04) and location (*χ*^2^ = 101.56, *p* ≤ 0.001) significantly influenced the frequency of antimicrobials usage. More men (57.9%) used antimicrobials regularly than women (42.1%). More women described their use of antimicrobials as occasionally (67.7%) and seldomly (85.7%) compared to men (occasionally: 32.3%, seldomly: 14.3%).

**Figure 1:**
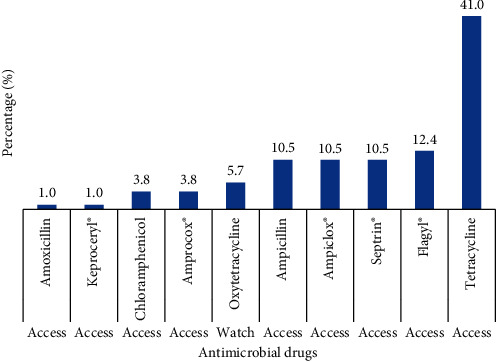
Antimicrobial drugs commonly used by farmers. Tetracycline is the most commonly used antimicrobial drug (*n* = 212).

**Figure 2:**
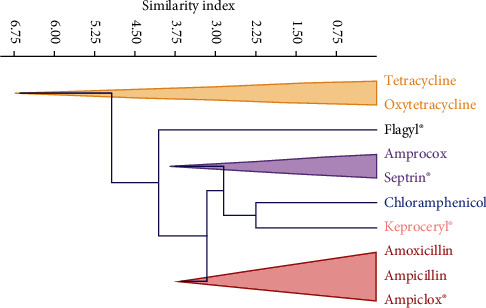
Cluster plot of the antimicrobial drugs used by farmers. Antimicrobials clustered as tetracyclines (tetracycline and oxytetracycline), sulfonamides (amprocox® and septrin®), penicillins (amoxicillin, ampicillin and ampiclox®), amphenicols chloramphenicol, and aminoglycosides (Keproceryl®).

**Figure 3:**
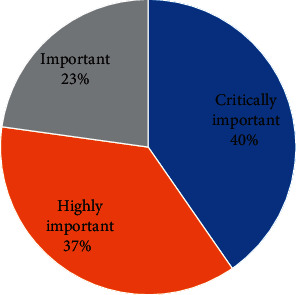
Categorisation of farmers' use of antimicrobials based on the WHO criteria for ranking antimicrobial drug use in human medicine (*n* = 212).

Over half (54.3%) of the farmers completed the required dosage during treatment compared to those (29.7%) who did not complete the dosage. Kebbi (50.0%) and Imo (46.0%) states accounted for most (98.4%) of the farmers who do not complete the required treatment dosage. Over one-tenth (16.0%) of the farmers did not know there was a required dosage for antimicrobials usage. A vast majority (87.3%) of the farmers stored the drugs somewhere in the house, with only a few (7.6%) using refrigerator. About one-third (31.6%) of the farmers knew the names of the antimicrobials administered. Most of the farmers (45.8%) did not know the names of the antimicrobials given to the birds but about one-fourth (22.6%) of these farmers could describe the antimicrobials.  Figure 1 shows the antimicrobial drugs commonly used by the farmers. All the antimicrobials, except oxytetracycline (watch list) were on the access group list of antimicrobials [20]. Tetracycline was the most commonly used antimicrobial drug by majority of the farmers (41.0%) while Keproceryl® (a mix of oxytetracycline, erythromycin, colistin and streptomycin) and amoxicillin were the least used antimicrobial drug by farmers (1.0%). Clustering of the drugs based on the similarity index for the different classes of antimicrobials showed the following clusters: Tetracycline and oxytetracycline (Tetracyclines), Amprocox® (Amprolium + sulphaquinoxaline sodium) and Septrin®/co-trimoxazole (Sulfonamides), amoxicillin, ampicillin and Ampiclox® (Ampicillin + Cloxacillin) (Penicillins), chloramphenicol (Amphenicols), Flagyl®/metronidazole (imidazole), and Keproceryl® (Aminoglycosides) (Figure 2). Further categorisation of the antimicrobials, based on the risk to human health was as follows: critically important (40%) (Ampiclox®, Keproceryl®, amoxicillin, ampicillin), highly important (37%) (Amprocox®, Septrin®, chloramphenicol, tetracycline, oxytetracycline), and important (23%) (Flagyl®) (Figure 3). The critically important antibiotic drugs were mostly used by farmers in Imo State (81.0%) compared to the other states (Nasarawa: 14.3%, Kebbi: 4.8%, Rivers and Kwara: 0%). In comparison with other states (Kebbi:0 and 9.4%, Nasarawa 7.1 and 17.2%, Rivers 7.1 and 1.6%, Kwara: 7.1 and 3.1%), Imo State represented the highest users of both the important (78.6%) and highly important (68.8%) antibiotic drugs. There were more women (Important: 64.3%, Highly important: 54.7%, Critically important: 71.4%) than men using all the three categories of antibiotics. Gender (*χ*2 = 1.99, *p* = 0.36) and location (*χ*2 = 6.54, *p* = 0.59) had no significant influence on antibiotics categorisation among the farmers.

### 3.6. Factors Associated with the Use of Antimicrobials

Table 6 highlights the influence of the independent factors (location, gender, education, age, income, farming experience, and management system) in predicting antimicrobial usage. The model shows that the factors were good predictors (*β* = 0.429, df = 1, *p* < 0.05) of antimicrobials use among the farmers. Educational level, family income, management system, age, location, and breed-type were 3.055, 1.987, 1.965, 0.741, 0.510, and 0.398 times more likely (*p* < 0.05) to influence farmers' use of antimicrobials, respectively. Gender, and farmers' years of keeping chicken did not have a statistically significant effect (*p* > 0.05) on the use of antimicrobials.

## 4. Discussion

Globally, the indiscriminate use of antibiotics in poultry production is a driver of antimicrobial resistance within the food chain. Antimicrobial use in smallholder poultry production in Nigeria is of particular importance due to the increasing shift in consumer preference for organically-raised village chickens (local, improved) produced under scavenging and semi-scavenging production systems. Chickens reared under such systems have been reported to have a lower risk of antimicrobial resistance [23, 24] because women who are the primary keepers of these birds, also serve as custodians of the indigenous veterinary knowledge (ethnoveterinary medicine) used in the treatment of diseases and general flock management [8, 10, 25, 26]. The result of this study agrees with previous studies on the role and dominance of women in smallholder poultry production in developing countries [26–28]. Compared to men (10.9%), over one-third (37.4%) of the women (37.4%) sampled in this study were uneducated (0 years of schooling). The high illiteracy observed among women limits the technical efficiency, productivity and performance of the primary chicken producers within the smallholder poultry value chain [29–31]. Specifically, it limits the capacity to adequately administer, monitor and keep records of antimicrobial use on the farm. This is supported by our findings on the record keeping, handling, storage and administration of antimicrobials by farmers sampled in this study.

High illiteracy among women also suggests that the women are innumerate. This portends a high possibility for the indiscriminate use of antimicrobials by resource-poor women, majority (70.3%) of whom earned below NGN 30,000 per month (i.e. USD 2.4/person/day). This is consistent with the overall poor outlook of women incomes and lower wages compared to men in smallholder farming [32]. Also, in a recent study, Bamidele and Amole [18] reported lower monthly incomes for women chicken producers in Nigeria compared to men.

Over half (54.0%) of the farmers had between 0 and 10 years' experience keeping smallholder poultry. This is similar to the number of years of farming experience reported by Alemayehu et al. [33], but is at variance with the report by Xu et al. [34] who observed that most farmers in Northwestern China had between 10 and 19 years farming experience. Despite the high percentage of farmers (83.1%) with over 5 years farming experience, a large proportion (88.9%) of the farmers had never been trained on poultry keeping, and only a few (11.1%) had received a form of training on animal health. Lack of basic training in husbandry and health management is a characteristic description of smallholder farming systems because farmers' access to quality training by subject-matter specialists and agricultural extension agents is poor and limited in rural communities [34, 35]. Keeping of improved and exotic chickens under the semi-scavenging and semi-intensive systems of production requires farmers to be adequately trained and informed on the management (housing, nutrition, health, biosecurity) skills applicable under such production systems [31]. Most farmers (39.4%) in this study, especially women practiced ethnoveterinary medicine (traditional method) for disease treatment and health management. This was most common with farmers who only kept local chickens (53.6%) as against those who reared exotic (42.0%) and improved (4.4%) chickens in addition to the local chicken ecotypes. Compared with the scavenging (16.0%) and semi-intensive (30.4%) production systems, the semi-scavenging system of production had the highest percentage (53.6%) of farmers applying the indigenous veterinary knowledge for bird treatment. Affordability (low cost) (38.4%) and availability (42.8%) were identified as the highest primary and secondary factors influencing the use of ethnoveterinary medicine, respectively. Previous studies have equally identified accessibility and low cost as the main drivers of ethnoveterinary medicine among farmers in SPPS [26, 36, 37]. Unsurprisingly, animal welfare, food safety and environmental considerations were the least factors motivating the use of traditional and local medicines on smallholder poultry farms.

Over two-thirds (35.4%) of the farmers were observed to be using a combination of ethnoveterinary and western methods of treatment in disease treatment and health management. This practice was common with farmers keeping exotic chickens (50.8%) in addition to local chickens than with those who kept improved (33.1%) breeds. It was least common with farmers who only kept local chickens (16.1%). While low cost of ethnoveterinary medicine was a major driver for its application, farmers who solely used western methods of pharmaceuticals treatment or in combination with traditional local herbs, indicated the cost implication of antimicrobial administration on the overall production and profitability of the farm enterprise. However, it has been reported that the cost of antimicrobials is relatively low and its use does not affect the profitability and economics of production in smallholdings [38, 39]. Administration of the antimicrobial drugs was mostly through water, a route previously reported as being the most used by poultry farmers [40–42].

This study observed a strong association between the locations (agroecology) and the various factors (primary and secondary) influencing the use of antimicrobials by farmers. These findings are in consonance with the reported intricate linkage between farmers' use of antimicrobials and veterinarians, poultry dealers and merchants, and sales representatives of feed companies and pharmaceuticals [43]. Our findings show that the behavioural tendency of farmers towards using antimicrobials is driven by the onset of any sign of illness in the birds, even though most of the farmers have not received any formal training on the identification of diseases and animal health management. This behaviour may explain the observed high percentage of farmers who indulge in self-prescription based on self-diagnosis, consequently risking an incorrect diagnosis and wrong use of antimicrobial drugs [44].

As previously reported by other studies for both commercial poultry (intensive) and small-scale poultry [32, 43–45], our findings also show multiple antimicrobial drug usage in SPPS. Multi-drug use was most common in semi-intensive production systems (69.7%) than in the other two systems (scavenging: 6.1%, Semi-scavenging: 24.2%), and was common with farmers who kept exotic (53.0%) and improved (33.3%) breeds. The high prevalence of multi-drug use observed in this study is similar to that previously reported for commercial and intensively managed farms in both developed and developing countries [32, 41, 46]. Unlike in developed nations, where policies have been enacted to prevent indiscriminate use of antimicrobials in livestock production, the ease of purchasing over-the-counter antimicrobial drugs, lack of training, and poor regulation of veterinary and animal practices are some of the factors fueling multi-drug use in the developing countries [6, 47]. Tetracycline in combination with either Flagyl® (24.2%) or Ampiclox® (18.2%) were the most predominant multi-drug used by farmers in this study. Tetracycline is reportedly one of the most commonly used antibiotics drug class in Africa [40, 43, 48].

All the antimicrobials were on the access list of antibiotic drug groups, except oxytetracyline, which was on the watch list. According to WHO [21], antibiotics on the access and watch lists have lower and higher resistance potentials than those on the reserve list (highest potential), respectively. This study identified the presence of the critically important (4 classes), highly important (5 classes), and important (1 class) antibiotic drugs used in human medicine [22]. The critically important antibiotic drugs were used by a larger proportion of the farmers (40%), than the other two classes. The presence of critically important antibiotics, with lower to higher resistance potential presents a potential public health threat, of antimicrobial resistance, to Nigerians who are increasingly demanding for organically-raised village chickens in place of the intensively-produced poultry products [49–51]. Our finding is consistent with the ranking of antimicrobial drug categorisation in Bangladesh, a country with similar economic and agricultural status as Nigeria [45].

The knowledge and awareness of farmers on the conditions (shelf-life, storage, withdrawal period, drug residue) for antimicrobial usage and the associated risks to humans, animals and the environment was poor. In addition to age and educational level, location, as a description of the agroecological zones was a significant predictor of antimicrobial usage among smallholder poultry farmers in Nigeria.

## 5. Conclusion

To the best of our knowledge, this study is the first to report on antimicrobial usage in SPPS across major agroecological zones of Nigeria. Most farmers still practice ethnoveterinary medicine. Antimicrobial drugs were used by farmers who kept both improved (FUNAAB Alpha, Noiler, Sasso, Kuroiler, ShikaBrown) and exotic (Broiler and layers) birds in addition to the local chicken ecotypes. Education, income and management system had the highest of influence on antimicrobial use. Multi-drug use was prevalent in the semi-intensive system of production with tetracycline being the drug mostly administered in combination with Flagyl® or Ampiclox®. There is a high risk of indiscriminate use of antimicrobial drugs within SPPS in Nigeria because of the high level of illiteracy observed among the women, who are the primary producers of village chickens. The use of critically important antibiotics threatens the consumption of poultry products, and presents smallholder poultry as a reservoir for antimicrobial resistance in humans. Provision of specialized trainings on animal disease and health management among smallholder poultry farmers will improve farmers' knowledge and awareness of antimicrobial resistance and stem the tide of antimicrobial drug abuse in livestock production.

## Figures and Tables

**Table 1:** Socio-demographic characteristics of Respondents.

Variable	Location						Statistic	*p* value
	Kebbi *n* = 70	Nasarawa *n* = 70	Rivers *n* = 70	Kwara *n* = 70	Imo *n* = 70	Total *N* = 350	*χ * ^2^ (df)	
Socio-demography								
*Gender*								
Male	16 (15.8)	7 (6.9)	20 (19.8)	23 (22.8)	35 (34.7)	101	29.14 (4)	*p* ≤ 0.001
Female	54 (21.7)	63 (25.3)	50 (20.1)	47 (18.9)	35 (14.1)	249		

*Education*								
None	36 (34.0)	20 (18.9)	22 (20.8)	28 (26.4)	0 (0.0)	106	97.108 (12)	*p* ≤ 0.001
Primary	9 (12.2)	11 (14.9)	25 (33.8)	21 (28.4)	8 (10.8)	74		
Secondary	12 (9.2)	33 (25.2)	17 (13.0)	18 (13.7)	51 (38.9)	131		
Tertiary	13 (33.3)	6 (15.4)	6 (15.4)	3 (7.7)	11 (28.2)	39		

*Age group*								
18–27	7 (58.3)	5 (41.7)	0 (0.0)	0 (0.0)	0 (0.0)	12	95.3 (20)	*p* ≤ 0.001
28–37	19 (42.2)	17 (37.8)	4 (8.9)	0 (0.0)	5 (11.1)	45		
38–47	15 (22.1)	15 (22.1)	13 (19.1)	13 (19.1)	12 (17.6)	68		
48–57	17 (14.7)	16 (13.8)	23 (19.8)	38 (32.8)	22 (19.0)	116		
58–67	10 (10.9)	17 (18.5)	27 (29.3)	18 (19.6)	20 (21.7)	92		
68 and above	2 (11.8)	0 (0.0)	3 (17.6)	1 (5.9)	11 (64.7)	17		

*Monthly income*								
<30,000	56 (26.4)	60 (28.3)	25 (11.8)	50 (23.6)	21 (9.9)	212	106.27 (8)	*p* ≤ 0.001
30,000–50,000	11 (9.4)	10 (8.5)	30 (25.6)	20 (17.1)	46 (39.3)	117		
>50,000	3 (14.3)	0 (0.0)	15 (71.4)	0 (0.0)	3 (14.3)	21		

values in parenthesis are percentages (%).

**Table 2:** Farmers knowledge and awareness of the implications and risk of antimicrobials.

Questions	Location					Total *N* = 212	*χ * ^2^ (df)	*p* value
	Kebbi *n* = 65	Nasarawa *n* = 41	Rivers *n* = 26	Kwara *n* = 28	Imo *n* = 52			
*Do you keep record of drugs used?*								
Yes	3 (4.7)	9 (14.0)	0 (0.0)	11 (17.2)	41 (64.1)	64	92.2 (4)	*p* ≤ 0.001
No	62 (41.9)	32 (21.6)	26 (17.6)	17 (11.5)	11 (7.4)	148		

*Are you aware of drug residue?*								
Yes	3 (25.0)	0 (0.0)	2 (16.7)	3 (25.0)	4 (33.3)	12	4.53 (4)	*p* ≤ 0.001
No	62 (31.0)	41 (20.5)	24 (12.0)	25 (12.5)	48 (24.0)	200		

*Are you aware of the withdrawal period when using antimicrobials and do you observe it?*								
Yes	1 (5.9)	1 (5.9)	7 (41.2)	2 (11.8)	6 (35.2)	17	18.93 (4)	*p* ≤ 0.001
No	62 (32.0)	40 (21.0)	19 (10.0)	26 (13.0)	46 (24.0)	195		

*Do you know that drugs have expiration/shelf-life?*								
Yes	2 (2.1)	7 (7.4)	25 (26.6)	9 (9.6)	51 (54.3)	94	148.01 (4)	*p* ≤ 0.001
No	63 (53.3)	34 (28.8)	1 (0.9)	19 (16.1)	1 (0.9)	118		

*Do you know if drugs taken by chickens pass to the eggs/meat?*								
Yes	1 (7.7)	1 (7.7)	7 (53.8)	3 (23.1)	1 (7.7)	13	25.50 (4)	*p* ≤ 0.001
No	64 (32.2)	40 (20.1)	19 (9.5)	25 (12.6)	51 (25.6)	199		

*Do you know if drugs in eggs/meat affect humans?*								
Yes	1 (7.7)	3 (23.0)	6 (46.2)	2 (15.4)	1 (7.7)	13	17.10 (4)	*p* ≤ 0.001
No	64 (32.2)	38 (19.1)	20 (10.0)	26 (13.1)	51 (25.6)	199		

*Do you know if there is any risk in eating/Selling of eggs/meat/chicken during or immediately after given drugs to the birds?*								
Yes	1 (9.1)	0 (0.0)	6 (54.5)	2 (18.2)	2 (18.2)	11	21.32 (4)	*p* ≤ 0.001
No	64 (31.8)	41 (20.4)	20 (10.0)	26 (12.9)	50 (24.9)	201		

*Are you aware of rules/regulations of antimicrobial usage?*								
Yes	1 (12.5)	0 (0.0)	3 (37.5)	0 (0.0)	4 (50.0)	8	10.12 (4)	0.04^∗^
No	64 (31.4)	41 (20.1)	23 (11.3)	28 (13.7)	48 (23.5)	204		

*Are you aware of any governing body responsible for controlling the use of antimicrobials in livestock production?*								
Yes	0 (0.0)	1 (33.4)	1 (33.3)	0 (0.0)	1 (33.3)	3	2.84 (4)	0.58
No	65 (31.1)	40 (19.1)	25 (12.0)	28 (13.4)	51 (24.4)	209		

*What do you consider as the most important risk to avoid?*								
Environmental pollution	1 (20.0)	0 (0.0)	0 (0.0)	4 (80.0)	0 (0.0)	5	40.41 (8)	*p* ≤ 0.001
Inappropriate usage causing harm to humans	0 (0.0)	6 (75.0)	0 (0.0)	2 (25.0)	0 (0.0)	8		
Loss of flock (mortality)	64 (32.2)	35 (17.6)	26 (13.1)	22 (11.0)	52 (26.1)	199		

*Are you aware that misuse and overuse of antimicrobials exposes humans and animals to antimicrobial resistant bacteria with dire consequence on human/animal health?*								
Yes	1 (6.7)	0 (6.7)	6 (40.0)	3 (20.0)	4 (26.6)	14	17.91 (4)	*p* ≤ 0.001
No	64(32.5)	41(20.3)	20(10.2)	25(12.7)	48(24.3)	198		

^*∗*^ *p*< 0.05, values in parenthesis are percentages (%).

**Table 3:** Features of the poultry production and management system.

Characteristics	Location						Statistics	
	Kebbi *n* = 70	Nasarawa *n* = 70	Rivers *n* = 70	Kwara *n* = 70	Imo *n* = 70	Total *N* = 350	*χ * ^2^ (df)	*p* value
*Farming experience (years)*								
1–5	19 (32.2)	4 (6.8)	3 (5.1)	0 (0.0)	33 (55.9)	59	120.47 (12)	*p* ≤ 0.001
6–10	36 (27.7)	24 (18.5)	34 (26.2)	14 (10.8)	22 (16.9)	130		
11–20	11 (10.8)	24 (23.5)	24 (23.5)	35 (34.3)	8 (7.8)	102		
>20	4 (6.8)	18 (30.5)	9 (15.3)	21 (35.6)	7 (11.9)	59)		

*General training on poultry husbandry and rearing*								
Yes	3 (7.7)	15 (38.5)	3 (7.7)	4 (10.3)	14 (35.9)	39	21.76 (4)	*p* ≤ 0.001
No	67 (21.5)	55 (17.7)	67 (21.5)	66 (21.2)	56 (18.0)	311		

*Specific training on animal diseases and health management*								
Yes	3 (18.8)	1 (6.2)	3 (18.8)	1 (6.2)	8 (50.0)	16	10.7 (4)	0.03^∗^
No	67 (20.1)	69 (20.7)	67 (20.1)	69 (20.7)	62 (18.6)	334		

*Type of chickens kept in addition to local chickens*								
Exotic	0 (0.0)	33 (23.9)	59 (42.8)	12 (8.7)	34 (24.6)	138	123.3 (4)	*p* ≤ 0.001
Improved	70 (52.2)	32 (23.9)	0 (0.0)	4 (3.0)	28 (20.9)	134		
None	0 (0.0)	5 (6.4)	11 (14.1)	54 (69.2)	8 (10.3)	78		

*Management system*								
Scavenging	29 (60.4)	0 (0.0)	15 (31.2)	1 (2.1)	3 (6.2)	48	154.54 (8)	*p* ≤ 0.001
Semi-scavenging	35(20.3)	46(26.7)	13 (7.6)	58(33.7)	20(11.6)	172		
Semi-intensive	6(4.6)	24(18.5)	42(32.3)	11(8.5)	47(36.2)	130		

^*∗*^ *p*< 0.05, values in parenthesis are percentages (%).

**Table 4:** Distribution of treatment methods available to farmers.

Characteristics	Location					Total *N* = 350	*χ * ^2^ (df)	*p* value
	Kebbi *n* = 70	Nasarawa *n* = 70	Rivers *n* = 70	Kwara *n* = 70	Imo *n* = 70			
*Treatment of chickens*								
Traditional	5 (3.6)	29 (21.0)	44 (32.0)	42 (30.4)	18 (13.0)	138	216.86 (8)	*p* ≤ 0.001
Western	60 (68.2)	1 (1.1)	0 (0.0)	15 (17.1)	12 (13.6)	88		
Traditional and western	5 (4.0)	40 (32.3)	26 (21.0)	13 (10.5)	40 (32.2)	124		

*Traditional method*								
	*n* = 5	*n* = 29	*n* = 44	*n* = 42	*n* = 18	*N* = 138		
*Primary reason for only using traditional method*								
Easily administered	0 (0.0)	8 (36.4)	4 (18.2)	10 (45.4)	0 (0.0)	22	50.35 (16)	*p* ≤ 0.001
Availability/accessibility	5 (12.5)	7 (17.5)	8 (20.0)	9 (22.5)	11 (27.5)	40		
Not costly	0 (0.0)	10 (18.9)	19 (35.8)	17 (32.1)	7 (13.2)	53		
Very effective	0 (0.0)	1 (5.3)	13 (68.4)	5 (26.3)	0 (0.0)	19		
Safe to birds, humans and the environment	0 (0.0)	3 (75.0)	0 (0.0)	1 (25.0)	0 (0.0)	4		

*Secondary reason*								
Easily administered	0 (0.0)	3 (12.5)	14 (58.3)	4 (16.7)	3 (12.5)	24	51.95 (12)	*p* ≤ 0.001
Availability/accessibility	0 (0.0)	20 (33.9)	11 (18.6)	22 (37.3)	6 (10.2)	59		
Not costly	5 (15.6)	4 (12.5)	4 (12.5)	12 (37.5)	7 (21.9)	32		
Very effective	0 (0.0)	2 (8.7)	15 (65.2)	4 (17.4)	2 (8.7)	23		
Safe to birds, humans and the environment	0 (0.0)	0 (0.0)	0 (0.0)	0 (0.0)	0 (0.0)	0		

values in parenthesis are percentages (%).

**Table 5:** Characteristics of farmers who used antimicrobials (western method alone, or in combination with traditional method).

Characteristics	Location					Total *N* = 212	*χ * ^2^ (df)	*p*value
	Kebbi *n* = 65	Nasarawa *n* = 41	Rivers *n* = 26	Kwara *n* = 28	Imo *n* = 52			
*Primary influence on the decision to use antimicrobials*								
Farmer group	0 (0.0)	3 (11.1)	15 (55.6)	0 (0.0)	9 (33.3)	27	320.19 (28)	*p* ≤ 0.001
Feed/drug seller	0 (0.0)	0 (0.0)	1 (9.1)	0 (0.0)	10 (90.9)	11		
Local merchants (day-old/brooded chicks)	20 (62.5)	2 (6.2)	0 (0.0)	10 (31.2)	0 (0.0)	32		
Neighbors/friend	1 (4.3)	3 (13.0)	3 (13.0)	7 (30.4)	9 (39.1)	23		
Veterinarian/animal health worker	44 (75.9)	4 (6.9)	1 (1.7)	3 (5.2)	6 (10.3)	58		
Own experiences	0 (0.0)	3 (8.8)	6 (17.6)	8 (23.5)	17 (50.0)	34		
Offtakers	0 (0.0)	0 (0.0)	0 (0.0)	0 (0.0)	1 (100)	1		
Extension agent	0 (0.0)	26 (100.0)	0 (0.0)	0 (0.0)	0 (0.0)	26		

*Secondary influence*								
Farmer group	5 (23.8)	0 (0.0)	5 (23.8)	0 (0.0)	11 (52.4)	21	163.5 (28)	*p* ≤ 0.001
Feed/drug seller	6 (15.4)	1 (2.6)	9 (23.1)	2 (5.1)	21 (53.8)	39		
Local merchants (day-old/brooded chicks)	10 (100)	0 (0.0)	0 (0.0)	0 (0.0)	0 (0.0)	10		
Neighbors/friend	7 (14.6)	16 (33.3)	7 (14.6)	9 (18.8)	9 (18.8)	48		
Veterinarian/animal health worker	33 (75.0)	3 (6.8)	5 (11.4)	3 (6.8)	0 (0.0)	44		
Own experiences	3 (7.0)	18 (41.9)	0 (0.0)	13 (30.2)	9 (20.9)	43		
Offtakers	0 (0.0)	3 (75.0)	0 (0.0)	1 (0.25)	0 (0.0)	4		
Extension agent	1 (33.3)	0 (0.0)	0 (0.0)	0 (0.0)	2 (66.7)	3		

*What has the most influence on the use of antimicrobials?*								
Birds are dying	21 (39.6)	0 (0.0)	21 (39.6)	4 (7.6)	7 (13.2)	53	83.69 (16)	*p* ≤ 0.001
The moment birds show any sign and symptom of disease	34 (28.1)	32 (26.4)	1 (0.8)	14 (11.6)	40 (33.1)	121		
Make birds eat/grow more	0 (0.0)	0 (0.0)	1 (100)	0 (0.0)	0 (0.0)	1		
Prevent sickness	10 (31.3)	9 (28.0)	3 (9.4)	10 (31.3)	0 (0.0)	32		
Prevent wastage of antimicrobials drug that is about to expire	0 (0.0)	0 (0.0)	0 (0.0)	0 (0.0)	5 (0.0)	5		

*Primary purpose of antimicrobials use*								
Treatment	10 (100)	0 (0.0)	0 (0.0)	0 (0.0)	0 (0.0)	10	49.35 (8)	*p* ≤ 0.001
Prevention	11 (30.6)	1 (2.8)	12 (33.3)	7 (19.4)	5 (13.9)	36		
Treatment and prevention	44 (26.5)	40 (24.1)	14 (8.4)	21 (12.7)	47 (28.3)	166		

*Source of prescription*								
Extension agent	56 (51.4)	39 (35.8)	0 (0.0)	2 (1.8)	12 (11.0)	109	173.98 (8)	*p* ≤ 0.001
Self-prescription	5 (7.1)	1 (1.4)	11 (15.5)	15 (21.1)	39 (54.9)	71		
Veterinarian/animal health worker	4 (12.5)	1 (3.1)	15 (46.9)	11 (34.4)	1 (3.1)	32		

*Source of drugs*								
Feedstore	3 (9.6)	25 (80.7)	0 (0.0)	1 (3.2)	2 (6.5)	31	209.25 (12)	*p* ≤ 0.001
Local vendor	43 (67.2)	1 (1.6)	0 (0.0)	5 (7.8)	15 (23.4)	64		
Pharmacy/Chemist	0 (0.0)	0 (0.0)	6 (13.3)	8 (17.8)	31 (68.9)	45		
Veterinary drug store	19 (26.4)	15 (20.8)	20 (27.8)	14 (19.4)	4 (5.6)	72		

*Has the COVID-19 pandemic affected how you source for these drugs?*								
Yes	59 (67.0)	25 (28.4)	4 (4.5)	0 (0.0)	0 (0.0)	88	171.56 (8)	*p* ≤ 0.001
No	5 (5.4)	11 (11.8)	9 (9.7)	28 (30.1)	40 (43.0)	93		
Not sure	1 (3.2)	5 (16.1)	13 (41.9)	0 (0.0)	12 (38.7)	31		

*Route of administration*								
Food	0 (0.0)	0 (0.0)	1 (11.1)	0 (0.0)	8 (88.9)	9	114.29 (12)	*p* ≤ 0.001
Water	50 (44.6)	5 (4.5)	21 (18.8)	27 (24.1)	9 (8.0)	112		
Food and water	15 (16.5)	36 (39.6)	4 (4.4)	1 (1.0)	35 (38.5)	91		
Injection	0 (0.0)	0 (0.0)	0 (0.0)	0 (0.0)	0 (0.0)	0		

Pattern of usage								
1 antimicrobial	50 (34.3)	39 (26.7)	26 (17.8)	13 (8.9)	18 (12.3)	146	61.93 (4)	*p* ≤ 0.001
≥2 antimicrobials	15 (22.7)	2 (3.1)	0 (0.0)	15 (22.7)	34 (51.5)	66		

*Frequency of antimicrobial use*								
Occasionally	65 (35.0)	37 (19.9)	25 (13.4)	11 (5.9)	48 (25.8)	186	101.56 (8)	*p* ≤ 0.001
Seldomly	0 (0.0)	4 (57.1)	1 (14.3)	1 (14.3)	1 (14.3)	7		
Regularly	0 (0.0)	0 (0.0)	0 (0.0)	16 (84.2)	3 (15.8)	19		

*Do you complete the required treatment dosage?*								
Yes	27 (23.5)	33 (28.7)	25 (21.7)	14 (12.2)	16 (13.9)	115	90.74 (8)	*p* ≤ 0.001
No	33 (52.4)	0 (0.0)	0 (0.0)	1 (1.6)	29 (46.0)	63		
Does not know there is a dosage	5 (14.7)	8 (23.5)	1 (3.0)	13 (38.2)	7 (20.6)	34		

*Storage of antimicrobials*								
Poultry shed	2 (22.2)	5 (55.6)	0 (0.0)	1 (11.1)	1 (11.1)	9	120.5 4 (12)	*p* ≤ 0.001
Refrigerator	0 (0.0)	1 (6.2)	15 (93.8)	0 (0.0)	0 (0.0)	16		
Somewhere in the house	63 (34.1)	35 (18.9)	11 (5.9)	27 (14.6)	49 (26.5)	185		
Outside the house	0 (0.0)	0 (0.0)	0 (0.0)	0 (0.0)	2 (100.0)	2		

*Do you know the name of the antimicrobials used?*								
Yes	6 (8.9)	11 (16.4)	2 (3.0)	2 (3.0)	46 (68.7)	67	143.44 (8)	*p* ≤ 0.001
No	49 (50.5)	12 (12.4)	21 (21.6)	11 (11.3)	4 (4.12)	97		
No, but I can describe it	10 (20.8)	18 (37.5)	3 (6.2)	5 (31.3)	2 (4.2)	48		

values in parenthesis are percentages (%).

**Table 6:** Distribution of independent variables predicting antimicrobial usage in smallholder poultry production.

Dependent variable: The use of antibiotics	*β*	SE	Wald statistics	Df	*p* value	OR	C.I. (OR)	
							Lower	Upper
*Constant*	1.115	0.993	1.261	1	0.261	3.048		
Location	−0.673	0.129	27.078	1	0.000^∗^	0.510	0.396	0.657
Gender	−0.389	0.343	1.291	1	0.256	0.678	0.346	1.326
Education	1.117	0.191	34.246	1	0.000^∗^	3.055	2.102	4.440
Age	−0.300	0.151	3.952	1	0.047^∗^	0.741	0.551	0.996
Family income	0.687	0.301	5.207	1	0.023^∗^	1.987	1.102	3.585
Years of keeping chickens	−0.150	0.167	0.802	1	0.371	0.861	0.621	1.195
Breed type	−0.922	0.391	5.567	1	0.018^∗^	0.398	0.185	0.855
Management system	0.676	0.287	5.532	1	0.019^∗^	1.965	1.119	3.452

^*∗*^ *p*< 0.05, *β*: beta coefficient, SE: standard error, Df: degree of freedom, OR: odds ratio, CI: confidence interval (95%).

## Data Availability

Data are available on request from the corresponding author. After institutional review, all data will be publicly available on the ILRI dataset portal at https://data.ilri.org/portal/.
